# Nitrogen-Doped Mesoporous Carbon Microspheres by Spray Drying-Vapor Deposition for High-Performance Supercapacitor

**DOI:** 10.3389/fchem.2020.592904

**Published:** 2020-11-10

**Authors:** Xiaoran Sun, Yueqi Kong, Yang Liu, Liang Zhou, Ashok Kumar Nanjundan, Xiaodan Huang, Chengzhong Yu

**Affiliations:** ^1^Institute of Photovoltaics, Southwest Petroleum University, Chengdu, China; ^2^Australian Institute for Bioengineering and Nanotechnology, The University of Queensland, Brisbane, QLD, Australia; ^3^School of Chemistry and Molecular Engineering, East China Normal University, Shanghai, China; ^4^State Key Laboratory of Advanced Technology for Materials Synthesis and Processing, Wuhan University of Technology, Wuhan, China

**Keywords:** mesoporous carbon, microspheres, spray drying, vapor deposition, supercapacitor

## Abstract

Nitrogen-doped mesoporous carbon microspheres have been successfully synthesized via a spray drying-vapor deposition method for the first time, using commercial Ludox silica nanoparticles as hard templates. Compared to freeze-drying and air-drying methods, mesoporous carbon with a higher packing density can be achieved through the spray drying method. Vapor deposition of polypyrrole followed by carbonization and etching is beneficial for the generation of ultra-thin carbon network. The mesoporous carbon microspheres possess a mesopore-dominate (95%) high surface area of 1528 m^2^ g^−1^, a wall thickness of 1.8 nm, and a nitrogen content of 8 at% in the framework. Benefiting from the increased apparent density, high mesopore surface area, and considerable nitrogen doping, the resultant mesoporous carbon microspheres show superior gravimetric/volumetric capacitance of 533.6 F g^−1^ and 208.1 F cm^−3^, good rate performance and excellent cycling stability in electric double-layer capacitors.

## Introduction

Electric double layer capacitors (EDLCs), a typical type of supercapacitors, are appealing power sources for consumer electronics and uninterruptible power supplies owing to their high power density, rapid charge/discharge, environmental friendliness, and long cycling life (Simon and Gogotsi, 2008, 2020; Choi et al., 2012; Wang et al., 2012; Liu et al., 2019, 2020; Noori et al., 2019; Li et al., 2020). Porous carbon materials have attracted tremendous attention as electrode materials for supercapacitors because of their high specific surface area, high conductivity, high chemical stability and tunable porous structures (Zhai et al., 2011; Tian et al., 2016, 2018; Hou et al., 2018; Wang et al., 2018; Liu et al., 2019; Shao et al., 2020). Microporous carbons with pore sizes smaller than 2 nm and high surface areas have been widely utilized in EDLCs (Zhang et al., 2009; Yan et al., 2014; Gadipelli et al., 2020). It was found that the capacitance reached a plateau when the microporous surface area went above 1,500 m^2^ g^−1^ (Barbieri et al., 2005; Weingarth et al., 2014), indicating that the total surface area measured by gas sorption methods is not fully electrochemically accessible. The partially inaccessible small micropores also decreased the rate performance of microporous carbon (Zhang and Zhao, 2009). In the past decades, mesoporous carbon materials with pore sizes of 2–50 nm have become promising candidates for EDLC applications (Simon and Gogotsi, 2008, 2020; Zhai et al., 2011; Choi et al., 2012; Wang et al., 2012, 2018; Tian et al., 2016, 2018; Hou et al., 2018; Liu et al., 2019, 2020; Noori et al., 2019; Li et al., 2020; Shao et al., 2020). Huang et al. (2008) demonstrated that the capacitance normalized by surface area was higher in mesoporous carbon compared to microporous carbon in aqueous solutions, suggesting a high capacitance can be achieved in carbon materials with high mesoporous surface area. Qian et al. reported the synthesis of mesoporous carbon with a high total surface area of 1,300 m^2^ g^−1^ and a high percentage of mesopore surface area (1,200 m^2^ g^−1^). This material showed a high capacitance of 340 F g^−1^ at 1 A g^−1^ and 66% capacity retention at 10 A g^−1^ due to the small pore size of 2–3 nm (Qian et al., 2014). Li et al. (2013) reported the fabrication of protein-derived carbons with a high mesopore surface area percentage of ~93% but a relatively small total surface area of 810 m^2^ g^−1^. There are few reports on the synthesis of mesoporous carbons with large mesopores and high mesopore surface areas (>1,400 m^2^ g^−1^) for supercapacitor applications.

In general, mesoporous carbons can be synthesized through soft-templating or hard-templating methods (Li et al., 2016). Surfactant molecules are generally used in the soft-templating method (Wei et al., 2013; Shen et al., 2015) and in the synthesis of mesoporous silicas as templates for the hard-templating approach (Ryoo et al., 1999). Instead of surfactants, using commercial Ludox silica nanoparticles as porogens is a convenient approach for the surfactant-free synthesis of mesoporous carbon (Han et al., 2000). However, the synthesis was usually conducted in solutions (Han and Hyeon, 1999), resulting in carbon materials with undefined morphology and relatively low packing density, unfavorable for the volumetric performance of EDLCs (Gogotsi and Simon, 2011; Wang et al., 2016). The packing density can be improved by controlling either the nanostructure or the micro-morphology (Li et al., 2015; Lin et al., 2016). Cui et al. reported the synthesis of porous carbon microspheres with increased packing density because small-sized spheres can be accommodated into the packing voids of large-sized spheres (Li et al., 2015). However, oil/water microemulsion was applied in the morphology control, not suitable for scalable synthesis. Spray drying, on the other hand, is a facile method for the synthesis of multi-shelled metal oxides (Zhou et al., 2013) and the assembly of colloidal silica nanoparticles into microspheres (Tan et al., 2012). When Ludox silica templates, resorcinol and formaldehyde as carbon precursors were used via the spray drying method, silica/polymer microspheres and subsequently mesoporous carbon microspheres were synthesized (Li et al., 2014), with a surface area below 1,200 m^2^ g^−1^. Wang et al. reported the synthesis of a partially graphitized porous carbon by spray drying Ludox silica with sucrose (Wang et al., 2015a,b). However, the capacitance of the obtained carbon microspheres is only 91 F g^−1^ at 10 mV s^−1^ due to the presence of a high proportion of micropores.

The choice of carbon precursors is crucial for replicating the template morphology with a homogenous carbon layer. Compared to resorcinol/formaldehyde or sucrose used in conventional infiltration and polyfurfuryl alcohol used in high-temperature chemical vapor deposition (CVD) (Lin et al., 2015), volatile precursors such as pyrrole applied in low-temperature vapor deposition achieved a uniform coating and maintained the structural stability of the template (Han et al., 2013). The high nitrogen content coming from carbonized polypyrrole contributed further to pseudo-capacitance and improved the overall capacitance of carbon materials (Shen and Fan, 2013; Wang et al., 2014). Ferrero et al. (2015) reported the preparation of N-doped hollow carbon nanospheres using vapor deposition of pyrrole on preformed silica nanoparticles, which have a faithfully replicated morphology of the silica template but a low packing density. It remains a challenge to use a convenient approach and synthesize mesoporous carbon microspheres with a high packing density, large mesopores and high mesopore surface areas for supercapacitors.

Herein, we report the preparation of nitrogen-doped mesoporous carbon microspheres by a spray drying-vapor deposition method for the first time, using commercial Ludox colloidal silica nanoparticles as porogens. The resultant mesoporous carbon microspheres have a mesopore-dominant (95%) high surface area (1,528 m^2^ g^−1^) with 8 At% nitrogen doping, an increased apparent density, and consequently excellent gravimetric/volumetric performance as an electrode material in EDLCs.

## Experimental Section

### Material Synthesis

Highly Nitrogen-doped mesoporous carbon microspheres were synthesized via spray drying and vapor deposition method. Typically, Ludox SM colloidal silica (40 g, 30 wt.% suspension in H_2_O, Sigma-Aldrich) was mixed with iron chloride (FeCl_3_) solution (0.3 M, 25 ml) and deionized water (200 ml). The mixture was ultrasonicated for over 1 h and kept stirring while spray drying. The suspension was spray dried at an inlet temperature of 220°C, a pump rate of 1.5 ml/min, and N_2_ gas flow of 60 ml/min. The spray dried sample was coated with pyrrole vapor for 48 h in 50°C oven before carbonized at 800°C for 10 h in a nitrogen atmosphere. Then the composite was washed with 5 wt.% hydrofluoric acid (HF) to remove the sacrificial silica template. The resulting product was centrifuged and washed repeatedly with deionized water and dried in an oven (addressed as MC-7-SD). For comparison, all other procedures remained the same, but the suspension was dried using 50°C oven or freeze-drier after sonication. The resultant was addressed as MC-7-AD or MC-7-FD, respectively. Activated carbon Darco® G-60 (-100 mesh, Sigma-Aldrich) was also used for comparison.

### Characterization

Field emission scanning electron microscopy (SEM) images were obtained using JEOL 7001. Transmission electron microscopy (TEM) images were taken using JEOL 1010 at 100 kV. High resolution transmission electron microscopy (HRTEM) was conducted under a Tecnai G2 F20 (FEI) operated at 200 kV. Nitrogen sorption isotherms were measured by Tristar II Surface Area and Porosity analyser (Micromeritics). The sample was degassed at 180°C overnight under vacuum before the test. X-ray photoelectron spectroscopy (XPS) was recorded on a monochromatic Al Kα (1,486.6 eV) X-ray source and 165 mm hemispherical electron energy analyzer. The elemental analysis was carried out using a Thermo Flash EA-1112 Series NCHS-O analyzer. Dynamic light scattering (DLS) measurement was conducted on a Malvern Zetasizer Nano ZS instrument at room temperature. Thermogravimetric (TG) analysis was performed using METTLER TOLEDO TGA/DSC1 STAR^e^ System from 25 to 900°C in air with a heating rate of 5°C min^−1^.

### Electrochemical Measurements

A slurry composing of active material (80 wt.%), carbon black (10 wt.%), and polytetrafluoroethylene (PTFE, Sigma-Aldrich, 60 wt.% dispersion in H_2_O) (10 wt.%) was mixed with ethanol. The working electrode was prepared by encapsulating mixture into Ni foam and dried at 100°C overnight. A three-electrode system was used to measure the electrochemical performance in 6 M KOH solution. Ni foam and Hg/HgO electrode were served as counter and reference electrode, respectively. Electrochemical measurements were carried out over a 1 V potential window (−1~0 V vs. Hg/HgO) using Solartron Multistat 1480. According to the capacitance value, the energy density (*E*) and power density (*P*) can be calculated using the following equations:

$$\begin{array}{l} E=CV^{2}/2 \end{array}$$

$$\begin{array}{l} P=E/t \end{array}$$

where *C* is the specific capacitance, *V* is the operating voltage, and *t* is the discharge time.

## Results and Discussion

**Figure 1:**
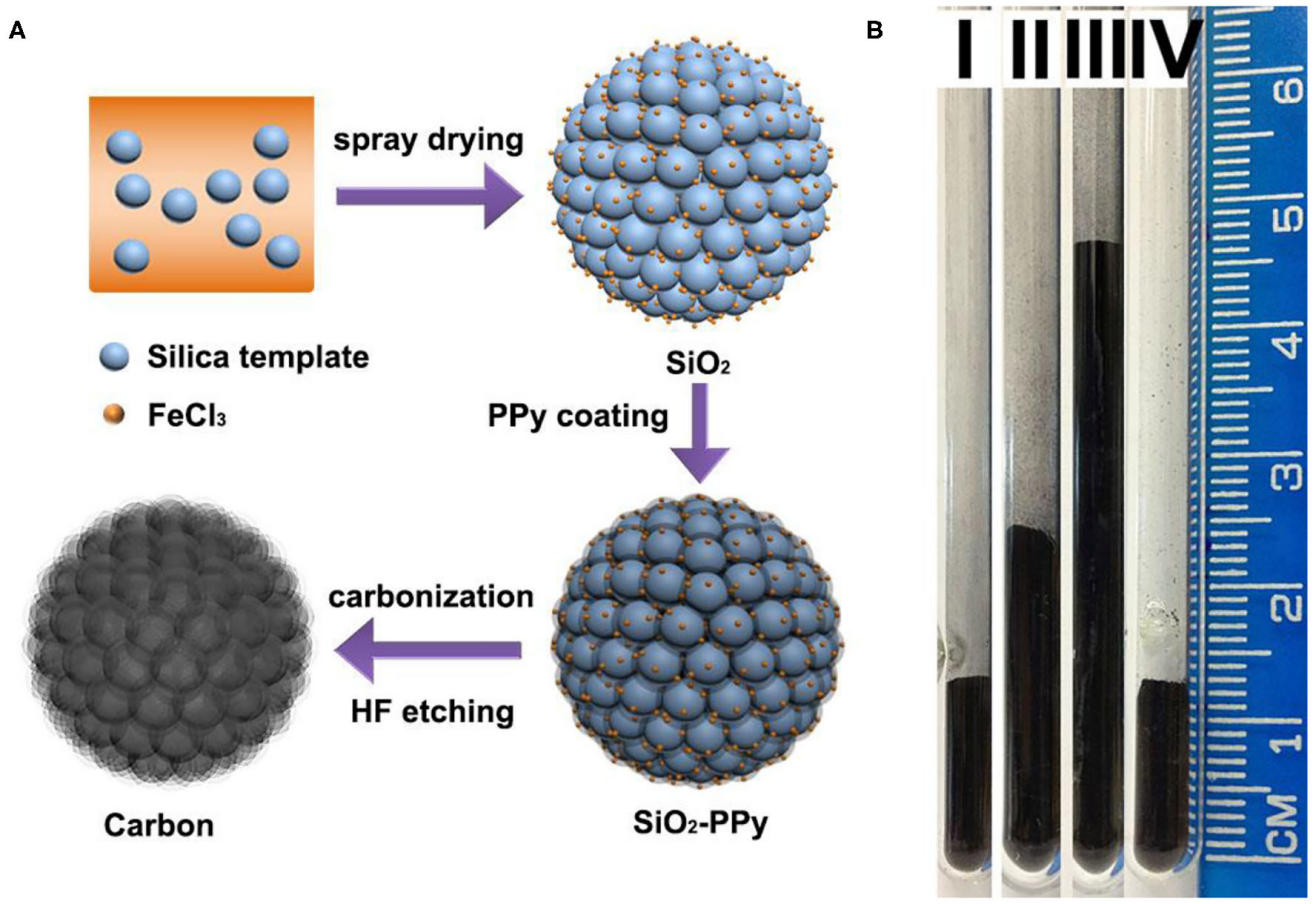
**(A)** Illustration of the synthesis approach of MC-7-SD, **(B)** the apparent density of (I) MC-7-SD, (II) MC-7-AD, (III) MC-7-FD, and (IV) activated carbon materials. The mass of each material is 50 mg.

Nitrogen-doped mesoporous carbon microspheres were synthesized by a spray drying-vapor deposition process (Figure 1A). Typically, commercially available colloidal silica nanoparticles (Ludox SM) with a diameter of around 7 nm (Supplementary Figures 1a,b) were dispersed in water. The suspension was atomized in the chamber of a spray drier to form droplets that were then dried in flight. Silica nanoparticles were agglomerated during the drying process, forming microspheres under contraction force. As a catalyst, iron chloride was added to the silica/water suspension and embedded in microspheres after spray drying for the oxidative polymerization of pyrrole. Then, pyrrole vapor was introduced to form a thin layer of polypyrrole coating on silica nanoparticles. After carbonization at 800°C in a nitrogen atmosphere and removing sacrificial silica template, N-doped mesoporous carbon microspheres were obtained (named as MC-7-SD). In order to highlight the advantage of spray drying method, silica aggregates were also synthesized using air-drying (Gierszal and Jaronic, 2007) and freeze-drying (Zhang et al., 2005) methods as reported in the literature for comparison. The resultant mesoporous carbons after 800°C carbonization and etching were denoted as MC-7-AD and MC-7-FD, respectively.

**Figure 2:**
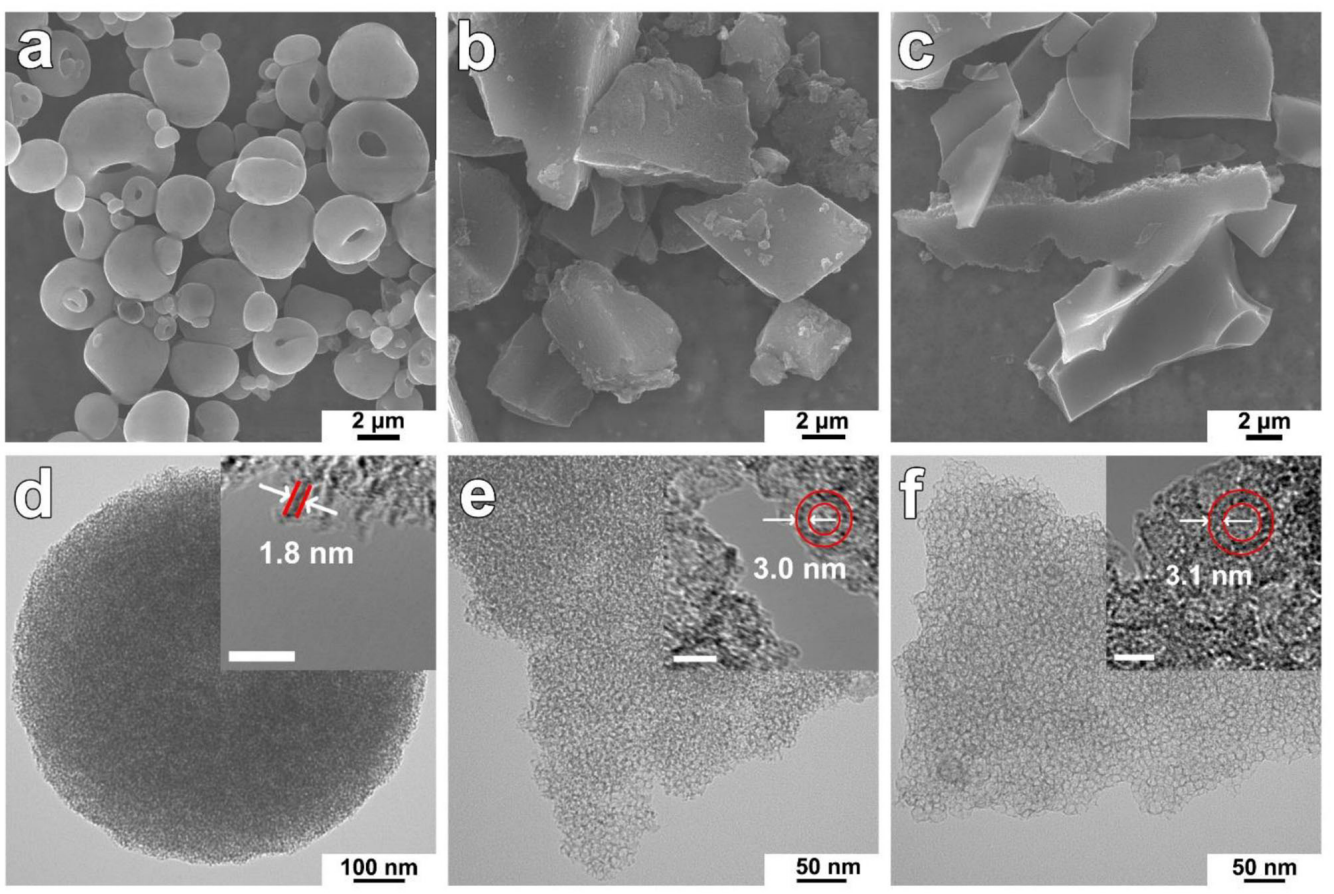
**(a–c)** SEM and **(d–f)** TEM images of MC-7-SD, MC-7-AD, and MC-7-FD, respectively. **(d–f)** insets are HRTEM images of the edge of MC-7-SD, MC-7-AD, and MC-7-FD, respectively. The scale bars in insets are 10 nm.

The apparent densities of MC-7-SD, MC-7-AD and MC-7-FD were measured as reported in the literature (Liu et al., 2016; Pei et al., 2016) and are shown in Figure 1B. MC-7-SD shows a higher apparent density (0.50 g cm^−3^) compared to MC-7-AD (0.31 g cm^−3^) and MC-7-FD (0.15 g cm^−3^), slightly lower than that of a commercial activated carbon (0.54 g cm^−3^). The obvious difference in apparent densities is explained in scanning electron microscopy (SEM) observations. The low magnification SEM image (Figure 2a) reveals that MC-7-SD has a micro-sized spherical morphology with a particle size of 700–5,000 nm (Supplementary Figure 1c). The invagination zones on the granules are probably caused by temperature (Biswas et al., 2016) and/or the polydispersity of constituent nanoparticles (Sen et al., 2012) during rapid spray drying. It is reported that the spherical morphology exhibits a higher packing density compared to other morphologies because the packing voids from large spheres accommodate smaller ones (Ying et al., 2004; Pan et al., 2020; Yue et al., 2020a,b). For comparison, MC-7-AD and MC-7-FD show fragmented morphologies with random particle packing (Figures 2b,c), leading to lower packing densities.

**Figure 3:**
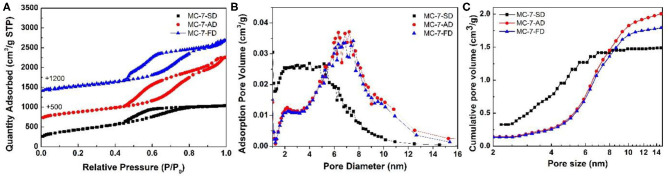
**(A)** Nitrogen adsorption/desorption isotherm, **(B)** pore size distribution curves and **(C)** cumulative pore volume-pore size plots of MC-7-SD, MC-7-AD, and MC-7-FD.

Transmission electron microscopy (TEM) was used to reveal the mesostructure of MC-7-SD, MC-7-AD, and MC-7-FD. TEM image of MC-7-SD (Figure 2d) shows a mesoporous micro-spherical morphology. As shown in Figure 2d inset, the thickness of the mesoporous carbon wall is around 1.8 nm. Different from MC-7-SD, the morphologies of MC-7-AD and MC-7-FD samples are monoliths with rich mesopores (Figures 2e,f). After measurement from the inset high magnification TEM images, the mesopore sizes in MC-7-AD and MC-7-FD are 7-8 nm, and the thicknesses of carbon layers are 3.0 and 3.1 nm, respectively. To fully characterize the porous structure of mesoporous carbon materials, nitrogen sorption analysis was employed. As shown in Figure 3A, all three samples show typical IV isotherms with H_2_ hysteresis loops. The adsorption branches of MC-7-AD and MC-7-FD show major capillary condensation steps in relative pressure (P/P_0_) range of 0.7–0.8 and >0.9, indicating the existence of large mesopores and packing voids. The adsorption isotherm of MC-7-SD shows a capillary condensation step at a relatively lower P/P_0_ of 0.6–0.75, indicating a relatively small pore size. No capillary condensation is observed at P/P_0_ > 0.9, suggesting small-sized spheres accommodates into the packing voids of large-sized spheres. The pore size distributions (PSD) calculated by Barrett–Joyner–Halanda (BJH) model from adsorption branches are shown in Figure 3B. The PSD of MC-7-AD and MC-7-FD show a peak centered at 7 nm, which is close to the size of Ludox silica templates (Supplementary Figures 1a,b). A shoulder peak at 2–5 nm can also be observed. Unlike MC-7-AD and MC-7-FD, MC-7-SD shows a wide PSD in the range of 2–9 nm, which can be attributed to the small mesoporous packing voids in the microspheres (Ying et al., 2004; Pan et al., 2020; Yue et al., 2020a,b).

As summarized in Table 1, the total pore volume of MC-7-AD, MC-7-FD, and MC-7-SD are 2.7, 2.3, and 1.6 cm^3^ g^−1^, respectively. MC-7-SD shows the lowest total pore volume because most of its pore volume is attributed by mesopores, while the total pore volumes of MC-7-AD and MC-7-FD are combinations of mesopores and packing voids. In order to differentiate the contribution of pore volume from mesopores with different sizes, the pore volume of specific pore size range is calculated from the cumulative pore volume-pore size plots (Figure 3C). In MC-7-SD sample, 55% of total pore volume (0.8 cm^3^ g^−1^) was attributed to mesopores with sizes of 2–5 nm, while only 20% of pore volume (0.3 cm^3^ g^−1^) came from mesopores with sizes of 5–9 nm (Table 1). On the contrary, 0.3 cm^3^ g^−1^ of pore volume of MC-7-AD and MC-7-FD was attributed to 2–5 nm mesopores (12 and 14%, respectively), and the majority of pore volume come from mesopores with sizes of 5–9 nm (1.3 cm^3^ g^−1^ (52%) and 1.2 cm^3^ g^−1^ (59%), respectively). MC-7-SD also shows the highest Brunauer-Emmett-Teller (BET) surface area (1,528 m^2^ g^−1^, Table 1) with an extraordinary mesoporous surface ratio (95%) compared to MC-7-AD and MC-7-FD (1,338 and 1,229 m^2^ g^−1^, respectively).

**Table 1:** Textural properties of MC-7-SD, MC-7-AD, MC-7-FD and activated carbon.

**Sample**	***S_***BET***_*** **(m^**2**^ g^**−1**^)**	***S_***micro***_*** **(m^**2**^ g^**−1**^)**	***S_***meso***_*** **(m^**2**^ g^**−1**^)**	***V_***t***_*** **(cm^**3**^ g^**−1**^)**	***V_**2−5*nm***_*** **(cm^**3**^ g^**−1**^)**	***V_**5−9*nm***_*** **(cm^**3**^ g^**−1**^)**
MC-7-SD	1,528	69	1,459	1.6	0.8	0.3
MC-7-AD	1,338	55	1,338	2.7	0.3	1.3
MC-7-FD	1,229	66	1,229	2.3	0.3	1.2
Activated carbon	840	498	342	0.8	–	–

*S_BET_ is BET surface area calculated from P/P_0_ = 0.05–0.3, S_micro_ is t-plot micropore area, S_meso_ is mesopore area calculated from equation S_meso_ = S_BET_-S_micro_, V_t_ is total pore volume at P/P_0_ = 0.995, V_a−bnm_ is the pore volume in the range of a–b nm, which is calculated from the cumulative pore volume*.

To investigate the formation of mesopores with sizes of 2–5 nm in MC-7-SD, MC-7-AD, and MC-7-FD, X-ray photoelectron spectroscopy (XPS) was conducted to analyze the Fe concentration on the surface of as-dried microspheres/aggregates. XPS spectra (Supplementary Figure 2) show the Fe/SiO_2_ molar ratios of the as-dried aggregates are 3.4 and 3.6% after air-drying and freeze-drying method, respectively, similar to the feeding ratio in silica/water suspension (3.7%). However, the Fe/SiO_2_ molar ratio of the as-dried microspheres after spray drying reduces to 1.1%, which is caused by the loss of Fe catalyst carried by inlet gas during spray drying (Wilkowska et al., 2016). As previously reported, the reduction of Fe catalyst results in less polypyrrole coated on the surface of spray-dried microspheres (Yang et al., 2005). The thermal gravimetric (TG) analysis of SiO_2_/C composites after carbonization (Supplementary Figure 3) further supported our conclusion. The composite after spray-drying method shows 9.5 wt.% carbon, lower than those after air-drying (12.9 wt.%) and freeze-drying methods (13.0 wt.%). The smaller carbon coating amount is in accordance with the thinner carbon layer observed from HRTEM. It is reported that thin carbon layer ends up in an invaginated morphology with reduced pore size after etching (Zhang et al., 2015). The nitrogen sorption results show that the size-reduced mesopores exist in MC-7-SD, MC-7-AD, and MC-7-FD. With a thinner carbon layer, MC-7-SD exhibits a higher proportion of small-sized (2–5 nm) mesopores compared to MC-7-AD and MC-7-FD.

In order to investigate the structural change of mesoporous carbon microspheres as a function of carbonization temperature, MC-7-SD was carbonized at 650, 800, and 950°C. As shown in Supplementary Figure 4, a typical mesoporous micro-spherical morphology is well maintained despite various temperatures. A slight increase of the specific surface area and pore volume was observed with the rising of carbonized temperature (Supplementary Table 1). The increase in specific surface area and pore volume is similar to a previous report (Shen et al., 2015), which is mainly due to the higher degree of carbonization at increased temperature. Raman spectra (Supplementary Figure 5) show the graphitization of mesoporous carbons. The distinguishable peak at 1,360 cm^−1^ (D band) is associated with the structural imperfections due to the lack of long-range order in amorphous and quasi-crystalline forms of carbon materials; meanwhile, the peak at 1,592 cm^−1^ (G band) corresponds to the E_2g_ species (stretching vibrations) of the infinite crystalline graphite (Tuinstra and Koenig, 1970; Wang et al., 1990; He et al., 2013). The intensity ratio between D and G band (I_D_/I_G_) provides a meaningful index for the crystallinity of carbon material. The I_D_/I_G_ ratio slightly decreases from 0.90 to 0.88 with the increase of the carbonization temperature from 650 to 950°C, which is attributed to the increasing crystallinity of carbon at higher carbonization temperature. The nitrogen doping contents of MC-7-SD samples carbonized at 650, 800, and 950°C were also investigated. The XPS survey spectra (Supplementary Figure 6) show the surface chemistry is dominated by C, O, and N for all mesoporous carbons. The N content decreases from 13.21 to 5.57 at% with the ascending temperature from 650 to 950°C. Elemental analysis (Supplementary Table 2) was conducted to determine the nitrogen content in bulk material. It is also shown that the nitrogen content in MC-7-SD samples decreases from 13.29 to 6.47 wt.% when the carbonization temperature increases from 650 to 950°C, which is attributed to the decomposition of N-containing functional groups at elevated temperatures (Shen et al., 2015). The capacitive performances of MC-7-SD carbonized at 650, 800, and 950°C were tested in 6 M KOH (Supplementary Figure 7). The charge-discharge curves of three samples exhibit a typical EDLC behavior with triangular shapes. As mentioned before, MC-7-SD carbonized at higher temperature possesses a better crystallinity, but the N-doping content is decreased. Consequently, the capacitance of MC-7-SD is the lowest (242.5 F g^−1^) carbonized at 650°C and the highest (362.8 F g^−1^) at 800°C. Further increasing the carbonization temperature to 950°C leads to decreased capacitance of 330.1 F g^−1^ (all at 1 A g^−1^). For this reason, 800°C was chosen as the optimized carbonization temperature of MC-7-SD, MC-7-AD, and MC-7-FD in the following studies.

**Figure 4:**
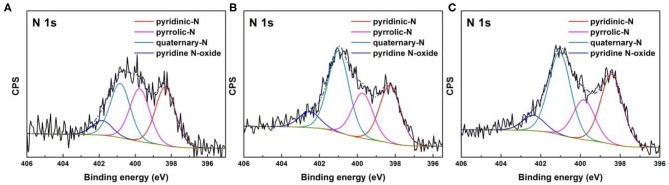
High-resolution N1s spectra of **(A)** MC-7-SD, **(B)** MC-7-AD, and **(C)** MC-7-FD.

Regardless of various drying method during synthesis, elemental analysis reveals that MC-7-SD, MC-7-AD, and MC-7-FD show similar nitrogen content after carbonization at 800°C (Table 2). XPS results further confirmed that about 8 at% nitrogen is accommodated in mesoporous carbons. Commonly, the high-resolution N1s spectra (Figure 4) include pyridinic-N (398.3 ± 0.1 eV), pyrrolic-N (399.7 ± 0.1 eV), quaternary-N (400.9 ± 0.1 eV), and pyridine N-oxide (402.2 ± 0.3 eV). Quaternary-N, also known as graphitic-N, has higher thermal stability by incorporating N atoms into graphitic carbon plane and bonded with three sp^2^ carbon atoms. As shown in Table 2, MC-7-SD, MC-7-AD, and MC-7-FD samples possess 30.09, 40.61, and 39.65% of quaternary-N, respectively. The high proportions of quaternary-N in polypyrrole-derived carbon are beneficial for the electron transfer and electrical conductivity of carbons (Hou et al., 2015). On the other side, pyrrolic-N and pyridinic-N often considered electrochemically active in aqueous solution, contributing pseudo-capacitance due to the additional p-electron donation to the aromatic π system (Lai et al., 2012; Sun et al., 2014). The total proportion of pyrrolic-N and pyridinic-N in MC-7-SD (32.54 and 29.23 at%) are higher than those in MC-7-AD (28.97 and 22.51 at%) and MC-7-FD (33.76 and 19.19 at%), possibly due to more edges in the carbon framework. With more electrochemically active nitrogen, MC-7-SD is expected to show larger pseudo-capacitive contribution in capacitance.

**Table 2:** The nitrogen-doping content of MC-7-SD, MC-7-AD, and MC-7-FD.

**Sample**	***N* (wt%)**	***N* (at%)**	**Pyridinic-*N* (at%)**	**Pyrrolic-*N* (at%)**	**Quaternary-*N* (at%)**	**Pyridine *N*-oxide (at%)**
MC-7-SD	9.46	8.67	32.54	29.23	30.09	8.14
MC-7-AD	9.75	7.05	28.97	22.51	40.61	7.91
MC-7-FD	9.33	8.50	33.76	19.19	39.65	7.40

**Figure 5:**
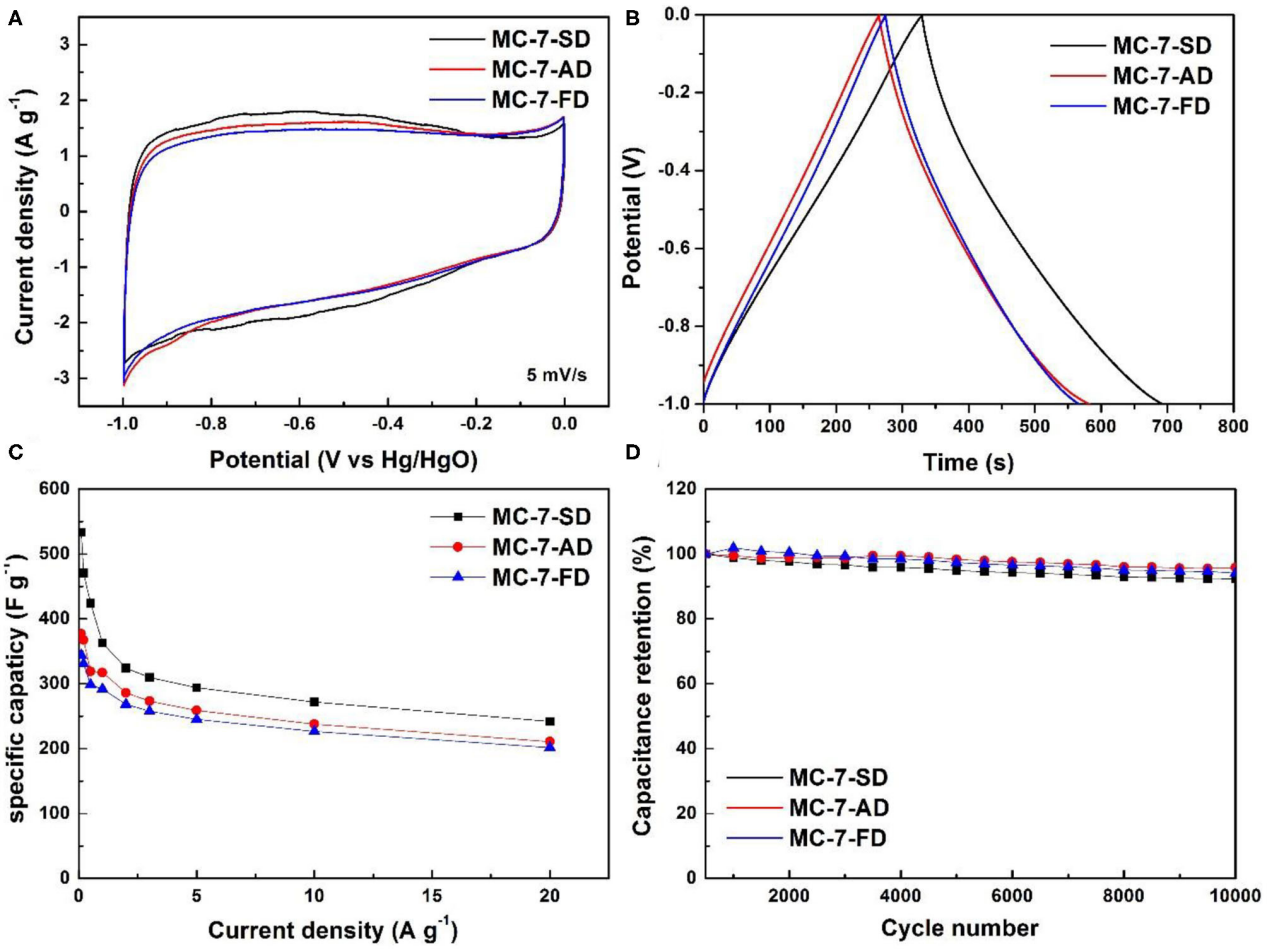
**(A)** Cyclic voltammetry curves at 5 mV s^−1^, **(B)** galvanostatic charge/discharge curves at 1 A g^−1^, **(C)** specific capacitance of MC-7-SD, MC-7-AD, and MC-7-FD and **(D)** cycle performance of MC-7-SD, MC-7-AD, and MC-7-FD at 5 A g^−1^.

The electrochemical performances of MC-7-SD, MC-7-AD, and MC-7-FD have been evaluated. Figure 5A shows the rectangular-shaped CV curves of MC-7-SD, MC-7-AD, and MC-7-FD. The broadened redox peaks in CV curves can be observed from −0.8 to −0.3 V. These wide and vague peaks are attributed to multiple faradaic peaks (Lin et al., 2015), partially associated with the pseudo-capacitance of pyrrolic-N/pyridinic-N (Xu et al., 2012). The representative galvanostatic charge-discharge curves of mesoporous carbons at a current density of 1 A g^−1^ are shown in Figure 5B. The charge-discharge curves are nearly linear and symmetric with a slight curvature, which suggests a good capacitive behavior and electrochemical reversibility (Zhang et al., 2016). The dependence of specific capacitances on the current density in Figure 5C shows that MC-7-SD delivers the highest specific capacitance (533.6 F g^−1^ at 0.1 A g^−1^, 362.8 F g^−1^ at 1 A g^−1^) with a high surface area normalized capacitance (23.74 μF cm^−2^ at 1 A g^−1^), giving a high maximum energy density of 74 Wh kg^−1^ (at 0.1 A g^−1^). The specific capacitance and surface area normalized capacitance of MC-7-SD are higher than those of micropore-dominate activated carbon (161.5 F g^−1^ and 19.22 μF cm^−2^ at 1 A g^−1^, Supplementary Figure 8), revealing the high utilization efficiency of mesopore-dominate structure. With higher specific surface area and more electrochemically active nitrogen, the capacitance of MC-7-SD (362.8 F g^−1^) is better than those of MC-7-AD and MC-7-FD (317.5 and 291.9 F g^−1^ at 1 A g^−1^, respectively). Electrochemical impedance spectroscopy (EIS) measurements of MC-7 supercapacitors were also performed. Typical Nyquist plots in the frequency range of 0.01 to 100,000 Hz are shown in Supplementary Figure 9. The Nyquist plot, composed of a depressed semicircle in high frequency region and a sloping line in low frequency area, can be fitted using an equivalent circuit (Supplementary Figure 9 inset). In the circuit, *R*_*e*_ represents the electrolyte and ohmic resistance, referring to the incept of the plot with *Z'* axis. *R*_*ct*_ and *Q* are the charge transfer resistance and double-layer capacitance, respectively, contributing to the depressed semicircle spanning *Z'* axis. The low frequency sloping line corresponds to the Warburg impedance (*Z*_*w*_) that pertains the ion diffusion in the electrode. Comparison of *R*_*ct*_ values for three MC-7 materials reveals the lowest *R*_*ct*_ of MC-7-SD, corresponding to improved charge transfer process due to efficient-packed carbon mesopores. After cycling, the plots maintained similar profiles.

It is noted the capacitance of MC-7-SD is comparable or better than those of mesoporous carbons reported in literature (Supplementary Table 3). Even at a high current density of 20 A g^−1^, a capacitance of 242.2 F g^−1^ (10 kW kg^−1^ power density) can be maintained, corresponding to 66.8% of capacity retention. Compared to other mesopore-dominant carbon materials (Qian et al., 2014), the higher capacity retention of MC-7-SD indicates the advantage of large mesopore sizes favorable for rapid charge/discharge. A long-lasting cycle life with <8% capacitance loss after 10000 cycles is obtained in MC-7-SD under 5 A g^−1^ (Figure 5D), slightly weaker than those of MC-7-AD and MC-7-FD (5 and 6% capacitance loss, respectively). This observation is in accordance with a previous report: larger pores facilitate the ion transportation, resulting in better cycling stability under high current charge-discharge (Ma et al., 2014). Apart from high gravimetric performance, MC-7-SD also achieves a superior volumetric capacitive behavior (208.1 F cm^−3^ at 0.1 A g^−1^) when calculating using an apparent density of 0.50 g cm^−3^. The superior electrochemical performance of MC-7-SD for EDLCs can be attributed to its unique structural features. Firstly, the thin carbon layer coated by vapor deposition results in a high mesoporous surface area, which leads to a high accessible electrode-electrolyte interface for electric double layer formation. Secondly, the high content of nitrogen doping provides additional electrochemically active nitrogen for pseudo-capacitance. Thirdly, relatively large mesopores in the carbon framework are beneficial for the remarkable capacitance retention at high current densities. Fourthly, the micro-spherical morphology of MC-7-SD is ideal for effective packing and excellent volumetric capacitive performance.

## Conclusion

In summary, nitrogen-doped mesoporous carbon microspheres have been successfully synthesized by spray drying-vapor deposition method. The resulting carbon microspheres possess a mesopore-dominate (95%) high surface area of 1528 m^2^ g^−1^, a nitrogen-doping of 8 at%, and an apparent density of 0.5 g cm^−3^. Benefiting from its unique features, MC-7-SD manifests excellent gravimetric/volumetric performance (533.6 F g^−1^ and 208.1 F cm^−3^). Our work opens up opportunities for the fabrication of efficient-packed porous carbon materials with heteroatom-doping for wide applications.

## Data Availability Statement

All datasets generated for this study are included in the article/Supplementary Material.

## Author Contributions

XS, LZ, XH, and CY designed the experiments. XS performed the material synthesis and electrochemical analysis. AN, YK, and YL performed material characterizations and electrochemical measurements. XS, XH, and CY co-wrote the manuscript. All the authors discussed the results, commented on the draft, and approved the final version of the manuscript.

## Conflict of Interest

The authors declare that the research was conducted in the absence of any commercial or financial relationships that could be construed as a potential conflict of interest.
