# Supplementary material for: Nitrogen-Doped Mesoporous Carbon Microspheres by Spray Drying-Vapor Deposition for High-Performance Supercapacitor
Source: Front Chem. 2020 Nov 10;8:592904. doi: 10.3389/fchem.2020.592904 (PMC7683435; doi:10.3389/fchem.2020.592904)
Supplement: Supplementary file 1 [file Table_1.DOCX]

Supplementary Material

Nitrogen-Doped Mesoporous Carbon Microspheres by Spray Drying-Vapor Deposition for High-Performance Supercapacitor

Xiaoran Sun^1,2^, Ashok Kumar Nanjundan^1^, Yueqi Kong^1^, Yang Liu^3^, Liang Zhou^4^, Xiaodan Huang^1*^ and Chengzhong Yu^1,3*^

Xiaoran Sun^1,2^, Yueqi Kong^2^, Yang Liu^3^, Liang Zhou^4^, Ashok Kumar Nanjundan^2*^, Xiaodan Huang^2*^ and Chengzhong Yu^2,3^

^1^Institute of Photovoltaics, Southwest Petroleum University, Chengdu 610500, P. R. China

^2^Australian Institute for Bioengineering and Nanotechnology, The University of Queensland, Brisbane, QLD 4072, Australia

^3^School of Chemistry and Molecular Engineering, East China Normal University, Shanghai 200241, P. R. China

^4^State Key Laboratory of Advanced Technology for Materials Synthesis and Processing, Wuhan University of Technology, Wuhan 430070, P. R. China


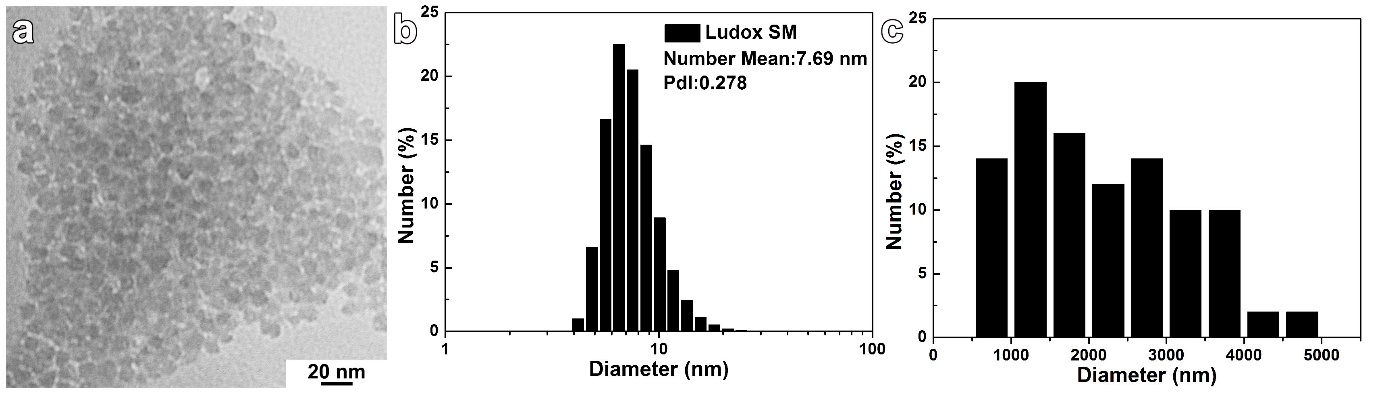


**Figure S1.** a) TEM image and b) DLS analysis of sacrificial silica template, c) Size distribution of N-doped mesoporous carbon microspheres.


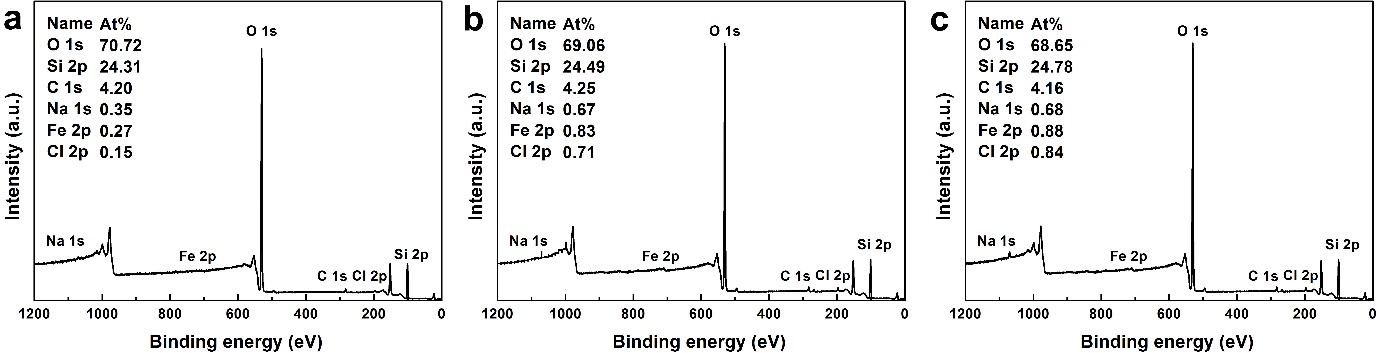


**Figure S2.** XPS survey spectra of a) MC-7-SD template, b) MC-7-AD template, and c) MC-7-FD template. The C comes from contamination and Na comes from the stabilizer in the pristine silica suspension.


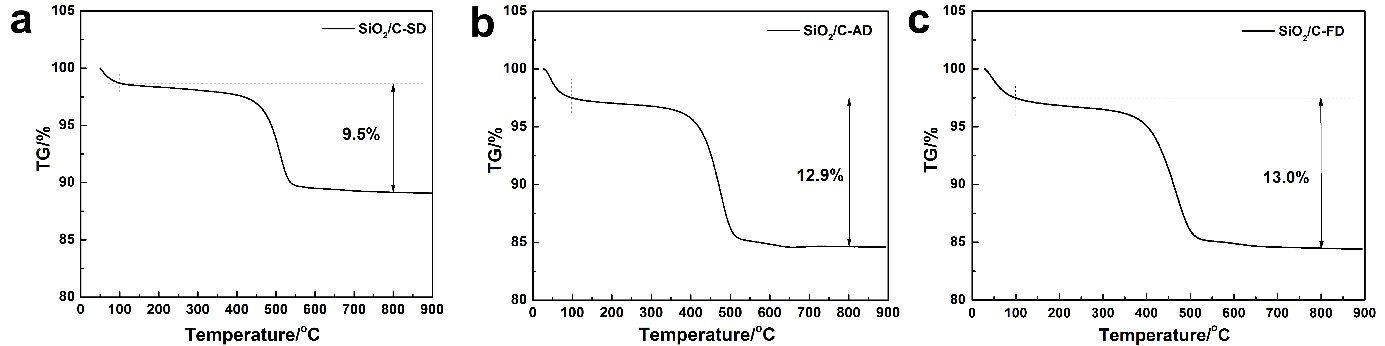


**Figure S3.** TGA spectra of silica/carbon composite synthesized using a) spray drying, b) air drying, and c) freeze drying method.


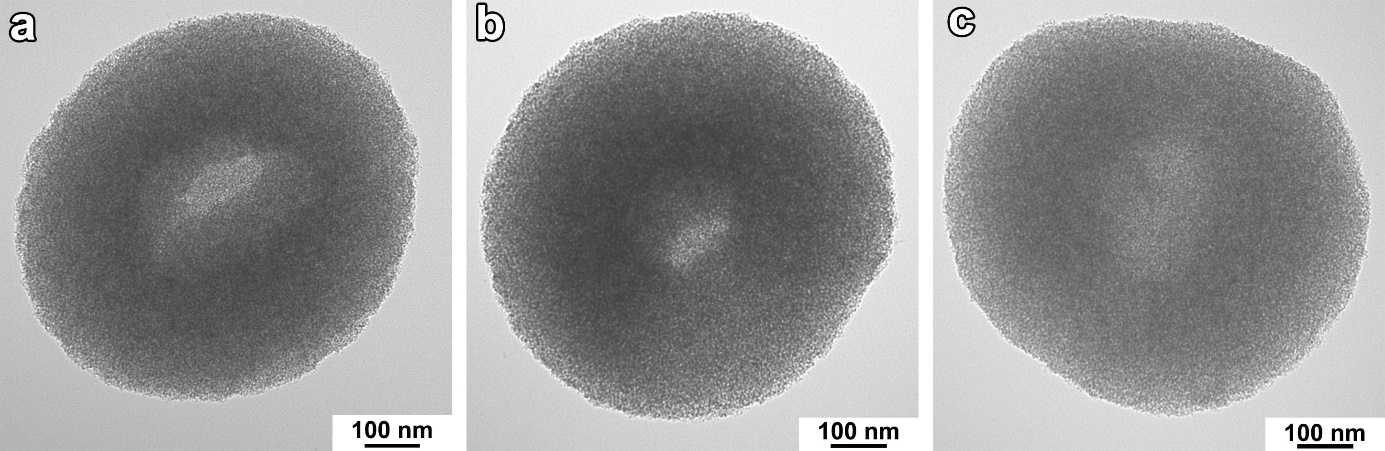


**Figure S4.** TEM images of MC-7-SD carbonized at a) 650 °C, b) 800 °C, and c) 950 °C.


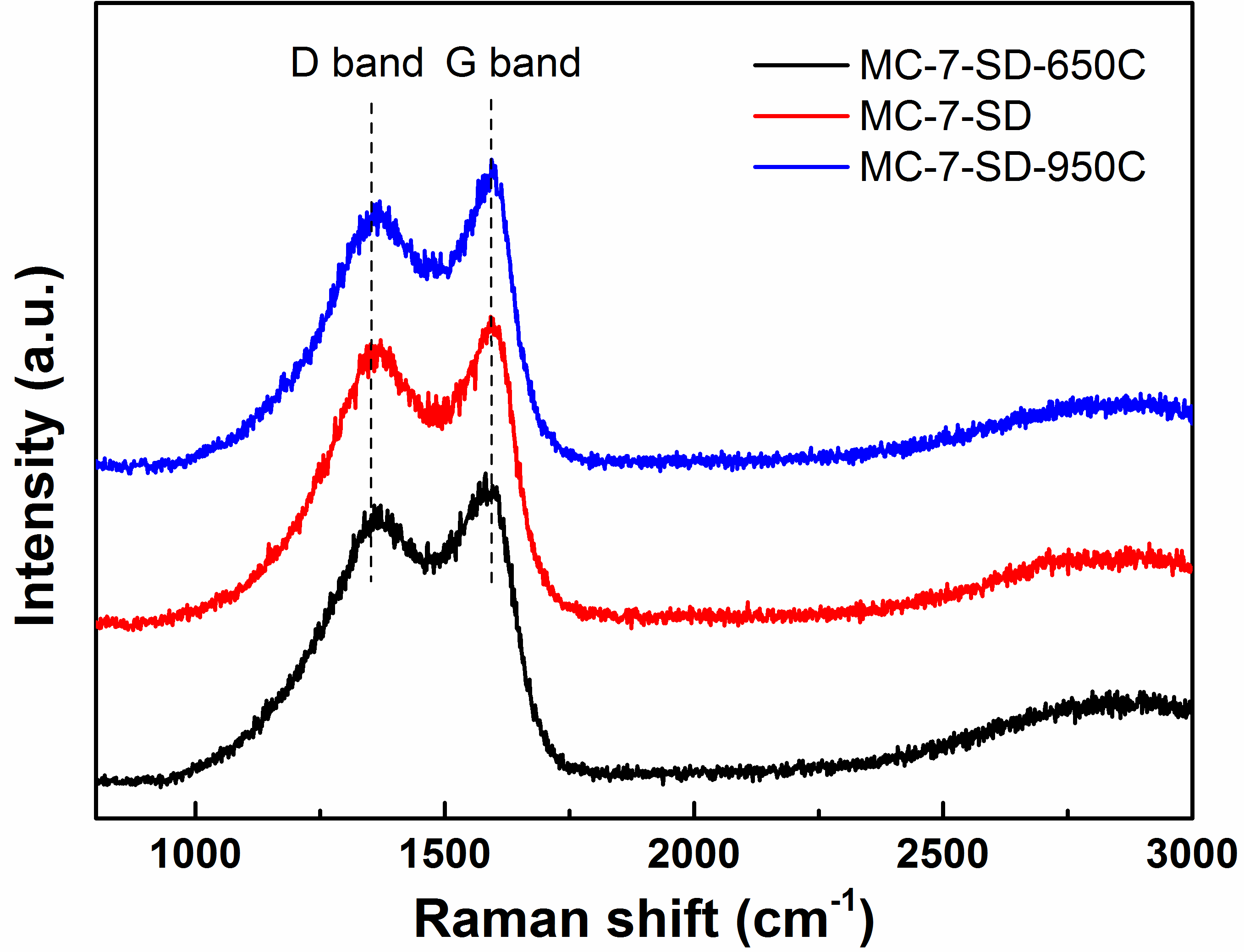


**Figure S5.** Raman spectra of MC-7-SD carbonized in different temperature.


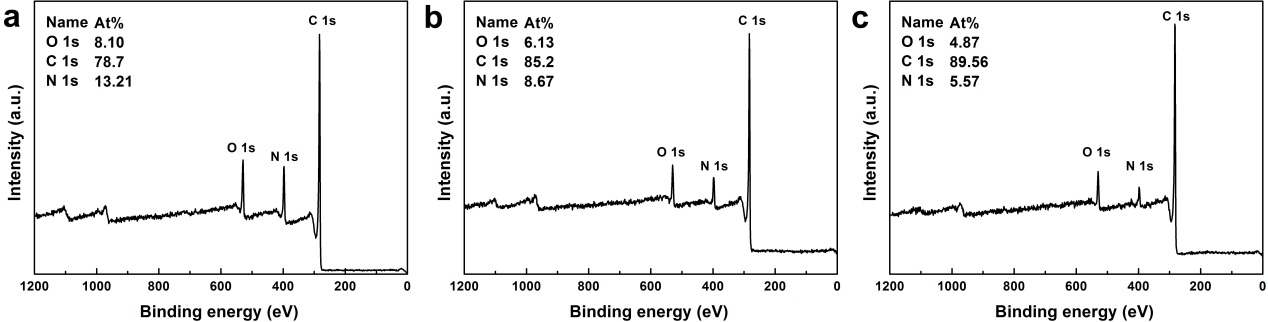


**Figure S6.** XPS survey spectra of MC-7-SD carbonized at a) 650 °C, b) 800 °C, and c) 950 °C.


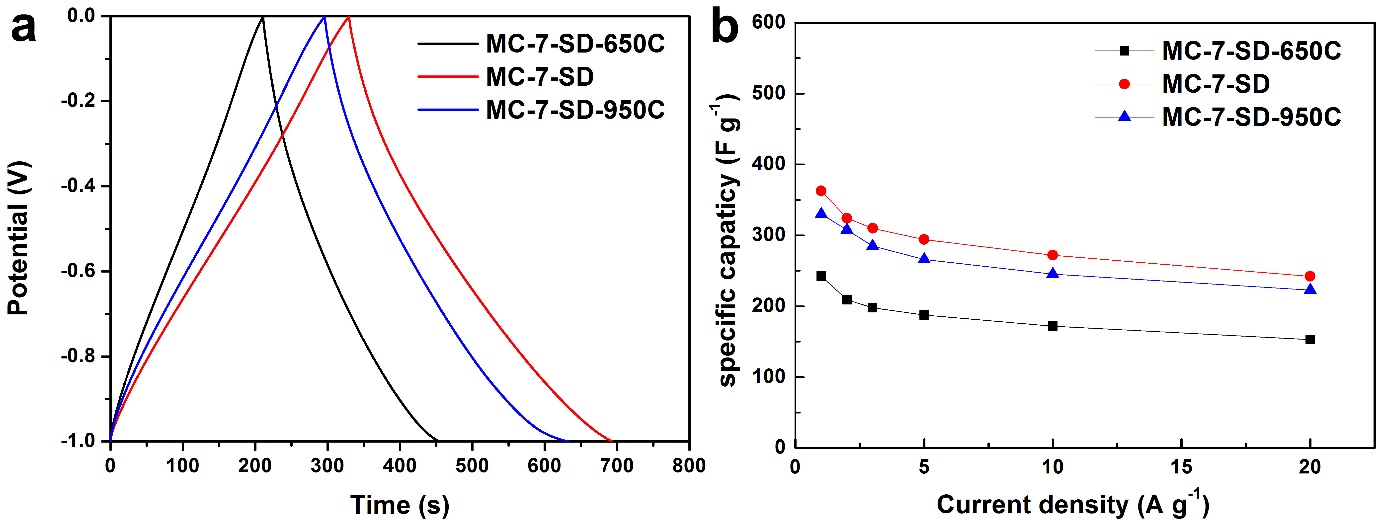


**Figure S7.** a) Galvanostatic charge/discharge curves at 1 A/g, b) specific capacitances of MC-7-SD carbonized in different temperature.


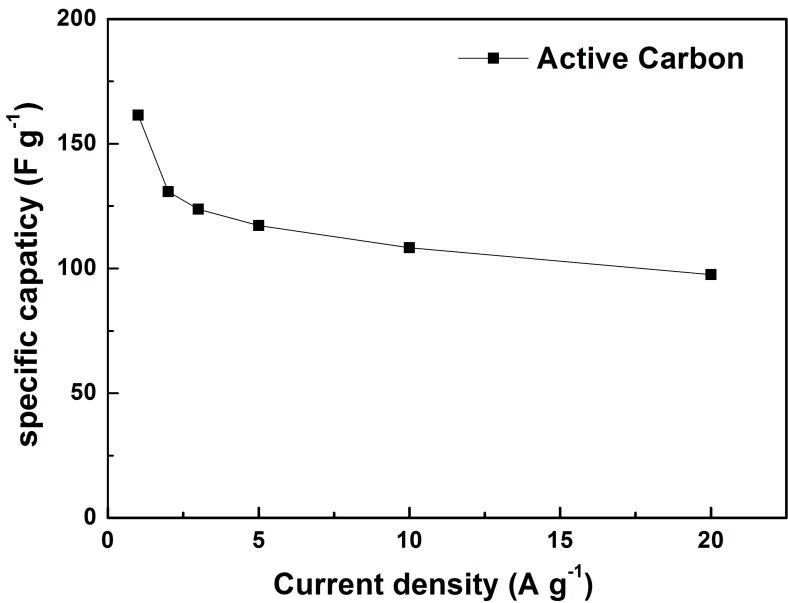


**Figure S8.** Specific capacitance of activated carbon.

**Table S1.** Textural properties of MC-7-SD carbonized in different temperature.

| Sample | *S_BET_* (m^2^ g^-1^) | *V_t_* (cm^3^ g^-1^) |
| --- | --- | --- |
| MC-7-SD-650C | 1437 | 1.4 |
| MC-7-SD | 1528 | 1.6 |
| MC-7-SD-950C | 1710 | 1.8 |

Note: *S_BET_* is BET surface area calculated from P/P_0_=0.05-0.3, *V_t_* is total pore volume at P/P_0_=0.995.

**Table S2.** Nitrogen-doping content of MC-7-SD carbonized in different temperature.

| Sample | MC-7-SD-650C | MC-7-SD | MC-7-SD-950C |
| --- | --- | --- | --- |
| Elemental analysis (wt%) | 13.29 | 9.46 | 6.47 |

**Table S3.** Electrochemical performance comparison among mesoporous carbon materials in aqueous electrolytes.

| Materials | Electrolytes | Specific capacitance | Reference |
| --- | --- | --- | --- |
| N-doped graphene nanosheets | 6 M KOH | 302 F g^-1^ at 5 mV s^-1^ | 1 |
| N-doped porous carbon nanofibers | 6 M KOH | 202 F g^-1^ at 1 A g^-1^ | 2 |
| N-doped ordered mesoporous carbon | 6 M KOH | 227 F g^-1^ at 0.2 A g^-1^ | 3 |
| 3D hierarchical porous carbon | 6 M KOH | 236.3 F g^-1^ at 2 A g^-1^ | 4 |
| N-enriched nanoporous carbon | 6 M KOH | 261 F g^-1^ at 0.02 A g^-1^ | 5 |
| Heteroatom doped porous carbon flakes | 6 M KOH | 340 F g^-1^ at 1 A g^-1^ | 6 |
| Functionalized graphene nanosheets | 6 M KOH | 456 F g^-1^ at 0.5 A g^-1^ | 7 |
| N-doped carbon nanocages | 6 M KOH | 313 F g^-1^ at 1 A g^-1^ | 8 |
| Hollow carbon nanospheres | 6 M KOH | 203 F g^-1^ at 0.1 A g^-1^ | 9 |
| Bamboo-loke carbon nanofiber | 3 M KOH | 236 F g^-1^ at 5 A g^-1^ | 10 |
| N-doped mesoporous carbon | 6M KOH | 405 F g^-1^ at 1 A g^-1^ | 11 |
| Mesoporous carbon hollow spheres | 6 M KOH | 310 F g^-1^ at 1 A g^-1^ | 12 |
| B/N co-doped carbon nanosheets | 6 M KOH | 223 F g^-1^ at A g^-1^ | 13 |
| Graphene nanoribbons | 1 M H_2_SO_4_ | 193 F g^-1^ at 10 mV s^-1^ | 14 |
| Hierarchical porous carbon nanosheets | 1 M H_2_SO_4_ | 233 F g^-1^ at 5 mV s^-1^ | 15 |
| Hierarchical micro-meso-macro carbon | 6 M KOH | 327 F g^-1^ at 0.2 A g^-1^ | 16 |
| Hierarchical porous carbon microrods | 6 M KOH | 406 F g^-1^ at 0.5 A g^-1^ | 17 |
| Rod-like activated porous carbon | 6 M KOH | 332 F g^-1^ at 1 A g^-1^ | 18 |
| Ultramicroporous carbons | 6 M KOH | 320 F g^-1^ at 0.5 A g^-1^ | 19 |
| **N-doped mesoporous carbon microspheres** | **6 M KOH** | **533.6 F g^-1^ at 0.1 A g^-1^** | **This work** |
|  |  | **362.8 F g^-1^ at 1 A g^-1^** |  |

**References**

[1] Wen, Z.; Wang, X.; Mao, S.; Bo, Z.; Kim, H.; Cui, S.; Lu, G.; Feng, X.; Chen, J., *Adv. Mater.* **2012**, *24*, 5610-5616.

[2] Chen, L.-F.; Zhang, X.-D.; Liang, H.-W.; Kong, M.; Guan, Q.-F.; Chen, P.; Wu, Z.-Y.; Yu, S.-H., *ACS Nano* **2012**, *6*, 7092-7102.

[3] Wei, J.; Zhou, D.; Sun, Z.; Deng, Y.; Xia, Y.; Zhao, D., *Adv. Funct. Mater.* **2013**, *23*, 2322-2328.

[4] Qie, L.; Chen, W.; Xu, H.; Xiong, X.; Jiang, Y.; Zou, F.; Hu, X.; Xin, Y.; Zhang, Z.; Huang, Y., *Energy Environ. Sci.*  **2013**, *6*, 2497-2504.

[5] Wu, D.; Li, Z.; Zhong, M.; Kowalewski, T.; Matyjaszewski, K., *Angew. Chem. Int. Ed.* **2014**, *53*, 3957-3960.

[6] Qian, W.; Sun, F.; Xu, Y.; Qiu, L.; Liu, C.; Wang, S.; Yan, F., *Energy Environ. Sci.* **2014**, *7*, 379-386.

[7] Yan, J.; Wang, Q.; Wei, T.; Jiang, L.; Zhang, M.; Jing, X.; Fan, Z., *Acs Nano* **2014**, *8*, 4720-4729.

[8] Zhao, J.; Lai, H.; Lyu, Z.; Jiang, Y.; Xie, K.; Wang, X.; Wu, Q.; Yang, L.; Jin, Z.; Ma, Y.; Liu, J.; Hu, Z., *Adv. Mater.* **2015**, *27*, 3541-3545.

[9] Xu, F.; Tang, Z.; Huang, S.; Chen, L.; Liang, Y.; Mai, W.; Zhong, H.; Fu, R.; Wu, D., *Nat Commun* **2015**, *6*, 7221.

[10] Sun, Y.; Sills, R. B.; Hu, X.; Seh, Z. W.; Xiao, X.; Xu, H.; Luo, W.; Jin, H.; Xin, Y.; Li, T.; Zhang, Z.; Zhou, J.; Cai, W.; Huang, Y.; Cui, Y., *Nano Lett.* **2015**, *15*, 3899-3906.

[11] Lin, T. Q.; Chen, I.-W.; Liu, F. X.; Yang, C. Y.; Bi, H.; Xu, F. F.; Huang, F. Q., *Science*, **2015**, *350*, 1508-1513.

[12] Zhang, H.; Noonan, O.; Huang, X.; Yang, Y.; Xu, C.; Zhou, L.; Yu, C., *ACS Nano* **2016**, *10*, 4579-4586.

[13] Ling, Z.; Wang, Z.; Zhang, M.; Yu, C.; Wang, G.; Dong, Y.; Liu, S.; Wang, Y.; Qiu, J., *Adv. Funct. Mater.* **2016**, *26*, 111-119.

[14] Pachfule, P.; Shinde, D.; Majumder, M.; Xu, Q., *Nat Chem* **2016**, *8*, 718-724.

[15] Jayaramulu, K.; Dubal, D. P.; Nagar, B.; Ranc, V.; Tomanec, O.; Petr, M.; Datta, K. K. R.; Zboril, R.; Gómez‐Romero, P.; Fischer, R. A., *Adv. Mater.* **2018**, *30*, 1705789.

[16] Qiu, Z. P.; Wang, Y. S.; Bi, X.; Zhou, T.; Zhou, J.; Zhao, J. P.; Miao, Z. C.; Yi, W. M.; Fu, P.; Zhuo, S. P., *Journal of Power Sources* **2018**, *376*, 82-90.

[17] Wu, F. M.; Gao, J. P.; Zhai, X. G.; Xie, M. H.; Sun, Y.; Kang, H. Y.; Tian, Q.; Qiu, H. X., *Carbon*, **2019**, *147*, 242-251.

[18] Jiang, W. C.; Pan, J. Q.; Liu, X. G., *J. Power Sources* **2019**, *409*, 13-23.

[19] Gadipelli, S.; Howard, C. A.; Guo, J.; Skipper, N. T.; Zhang, H.; Shearing, P. R.; Brett, D. J. L., *Adv. Energy Mater.* **2020**, *10*, 1903649
